# Recurrent Hypoglycemia in a Case of Congenital Analbuminemia

**DOI:** 10.1155/2020/8452564

**Published:** 2020-02-27

**Authors:** Martin Litzel, Gianluca Caridi, Francesca Lugani, Monica Campagnoli, Lorenzo Minchiotti, Stefan Fischli

**Affiliations:** ^1^Division of Endocrinology, Diabetes and Clinical Nutrition, Luzerner Kantonsspital, Luzern 6000, Switzerland; ^2^Laboratory of Molecular Nephrology, Istituto Giannina Gaslini, IRCCS, Genoa 16147, Italy; ^3^Department of Molecular Medicine, University of Pavia, Pavia 27100, Italy

## Abstract

In congenital analbuminemia (CAA), mutations in the albumin gene result in a severe deficiency or absence of plasma albumin. Only about 90 cases have been reported to date, but the specific features of glucose and lipid metabolism in congenital analbuminemia have only been studied in a rat model of analbuminemia. We report the case of a female patient hospitalized for a streptococcal skin infection who showed recurrent hypoglycemia. A diagnosis of CAA was confirmed by mutation analysis and by the detection of a single base variation in the *ALB* gene. Hypoglycemia was first documented after a fasting period during acute illness. Recurrent hypoglycemia persisted despite good general condition and normal nutrition during antimicrobial therapy with moxifloxacin. Several contributing factors causing this hypoglycemia can be discussed. Individuals with CAA are prone to adverse drug effects caused by changes in drug-protein binding properties. It is unclear if specific changes of glucose and lipid metabolism in CAA constitute a risk factor for hypoglycemia.

## 1. Background

Congenital analbuminemia (CAA; OMIM # 616000) is a rare, autosomal recessive inherited disorder that is usually characterized by mutations in the albumin gene (*ALB*; NCBI Genomic Sequence: NC_00004.12), which lead to undetectable or low levels of the circulating protein [1]. Despite the condition being easily detected by routine serum protein electrophoresis, it is very rare, with only ∼90 cases having so far been identified worldwide [1]. Thus, the estimated prevalence is <1 in 1 million [1]. CAA is relatively well tolerated in adulthood but can lead to serious consequences in the pre- and perinatal periods, during which it can cause preterm birth or neonatal or early childhood death [1]. Typical clinical findings in CAA include lower extremity edema and lipodystrophy, which are more obvious in women, and orthostatic hypotension. CAA should be suspected in the presence of a low or undetectable plasma albumin concentration in the absence of renal or gastrointestinal albumin loss or hepatic disease. Electrophoresis of plasma proteins characteristically shows a small albumin fraction and larger alpha-1, alpha-2, beta, and gamma fractions. Nonalbumin plasma proteins, such as immunoglobulins, coagulation factors, hormone-binding proteins, and lipoproteins, are present in higher concentrations as a compensatory mechanism. Hypercholesterolemia, with a high concentration of low-density lipoprotein (LDL) cholesterol, is a typical finding, but it is as yet unclear whether the dyslipidemia in CAA is associated with higher cardiovascular morbidity and mortality. Hypercholesterolemia can be reversed by administration of albumin indicating an interplay between albumin and lipid metabolism [2]. Total serum calcium is usually low, while ionized calcium is in the normal range, and symptomatic hypocalcemia has not been described in CAA [1].

The effects of analbuminemia on glucose and lipid metabolism have previously been studied in a rat model. Analbuminemic rats (NAR) were intolerant of carbohydrate deprivation and showed earlier mortality than normal rats (NR) during fasting [3]. One recognized mechanism is an impairment in lipid metabolism, associated with free fatty acid (FFA) deficiency. Normally, fasting induces gluconeogenesis, glycogenolysis, ketogenesis, and FFA oxidation. However, FFAs in plasma are largely bound to albumin, and an albumin deficiency causes lower intravascular lipolysis as well as reductions in FFA availability and transportation to target tissues. However, albumin administration to NAR reduces this FFA deficiency [4]. In addition, glucose metabolism in NAR is affected. In comparison to NR, glycogenolysis, as well as gluconeogenesis in albumin-deficient NAR is upregulated. Liver glycogen after a fasting period of 6 or 14 hours was significantly lower in NAR compared to NR. The injection of a gluconeogenic substrate caused a faster rise of glucose concentrations in NAR compared to NR. Glucose tolerance tests showed a more rapid return of glucose concentration to normal levels in the analbuminemic rats. These findings could be attributed to an enhanced islet responsiveness with increased insulin secretion of NAR compared to NR [5].

Individuals with CAA are vulnerable to the adverse effects of drugs that have high protein-binding affinities and that are acidic due to alterations in the pharmacokinetics and bioavailability of these drugs [1]. Hypoglycemia is a recognized side effect of fluoroquinolones, such as moxifloxacin, and is due to a blockade of pancreatic islet ion channels, which causes an increase in insulin secretion [6, 7]. A large proportion of moxifloxacin is bound to plasma proteins and specifically to albumin [8].

## 2. Case Report

### 2.1. Clinical Findings

A 40-year-old woman with a previous clinical diagnosis of CAA [9] was hospitalized for severe lower leg cellulitis. The cellulitis was diagnosed by her general physician five days before and treated with oral amoxicillin/clavulanic acid. She reported redness and pain in both lower legs of 6 days' duration. Because of general sickness, nausea, and loss of appetite, food intake was reduced to a minimum since six days. Clinical examination revealed obesity (height: 158 cm; weight: 100 kg; body mass index: 40 kg/m^2^), normal vital signs (temperature: 36.5°C; blood pressure: 111/67 mmHg; heart rate: 91 bpm), and bilateral lipodystrophy of the lower extremities, with edema, tenderness, redness, and warmth.

**Figure 1:**
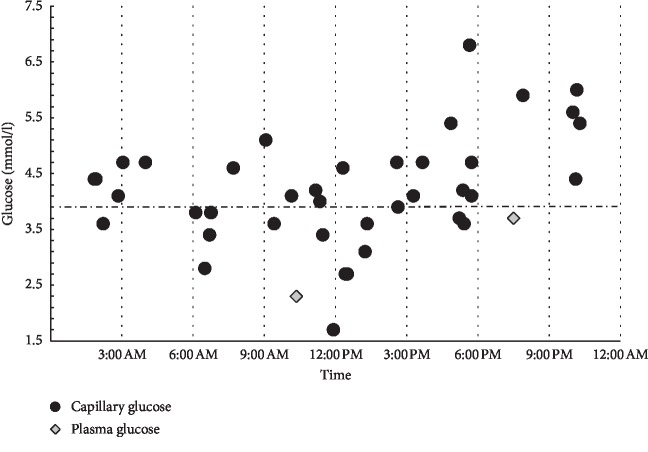
Capillary and plasma glucose values that were recorded during 7 days of hospitalization. All values are projected to a 24-hours view. These values were recorded on the first presentation at hospital admission. The dashed line marks the cutoff value for hypoglycemia with 3.9 mmol/l.

A clinical diagnosis of CAA was proposed when the patient was 23 years old and hospitalized for treatment of a popliteal deep vein thrombosis and lower leg cellulitis [9]. Capillary serum protein electrophoresis confirmed the near-complete absence of the albumin peak and a compensatory increase in the other protein fractions (data not shown). Her family history was suggestive of analbuminemia in her younger brother and mild hypoalbuminemia in her older brother, suggesting a heterozygous state [9]. Her personal history included recurrent deep vein thrombosis, severe bilateral lower limb lipedema, and dyslipidemia. Liposuction had been performed several times on both legs and had been complicated by postoperative cellulitis. Her current medication consisted of oral amoxicillin/clavulanic acid, rivaroxaban, diclofenac, and a statin/ezetimibe combination. With hospital admission, oral amoxicillin/clavulanic acid was replaced by intravenous application, and clindamycin was added two days later. Because of poor response of the cellulitis to the previous therapy, piperacillin/tazobactam was initiated and amoxicillin/clavulanic acid and clindamycin was stopped after 15 days. At the initial in-hospital examination, capillary glucose measurement identified severe hypoglycemia (1.7 mmol/l). Despite oral administration of dextrose, recurrent low-glucose concentrations were identified while the patient was hospitalized (Figure 1). However, her cellulitis and general condition improved gradually, and she was discharged after 3 weeks. The antimicrobial treatment was then switched to oral moxifloxacin.

**Figure 2:**
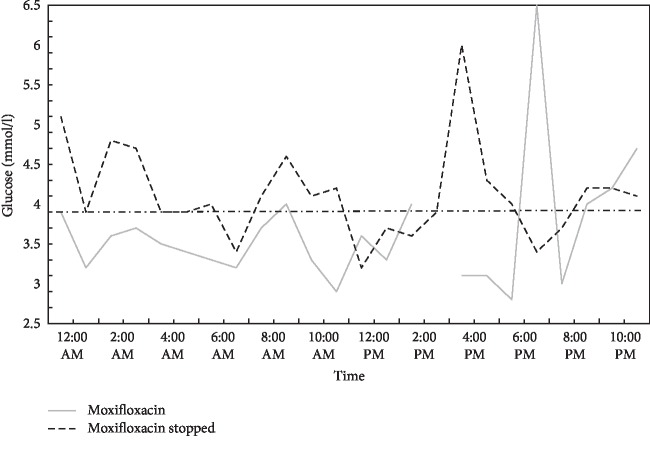
Hourly glucose concentration minima obtained using the sensor flash-glucose monitoring system during treatment with moxifloxacin and 4 weeks after it was stopped. The lines show the lowest glucose levels that were recorded for each hour. Each monitoring period covered 14 days. The dashed line marks the cutoff value for hypoglycemia with 3.9 mmol/l.

The patient then attended the endocrine outpatient clinic for evaluation of the recurrent hypoglycemia. Glucose monitoring using a flash-glucose monitoring system (FreeStyle libre®, Abbott Diabetes Care, Alameda, California) over 14 days confirmed frequent hypoglycemia, with a minimum of 2.8 mmol/l (Figure 2). The patient never showed signs of neuroglycopenia. Renal or hepatic insufficiency and hypocortisolism were excluded (Table 1a), and she was not using any hypoglycemic drugs. One month after the discontinuation of moxifloxacin, continuous flash-glucose monitoring was repeated, which showed an increase in her glucose concentrations, but there were still several values close to or below the lower limit of the normal range (Figure 2). In addition, a 10 h fasting test was performed, but her plasma glucose, free fatty acids, and hydroxybutyrate concentrations were normal (Table 1b). However, the levels of free fatty acids were clearly lower than in a comparable reference group of fasting obese subjects without insulin resistance [10]. In a follow-up conversation 8 months later, the patient reported general well being. There were no possible symptoms of hypoglycemia.

### 2.2. Mutation Analysis of the ALB Gene

**Figure 3:**
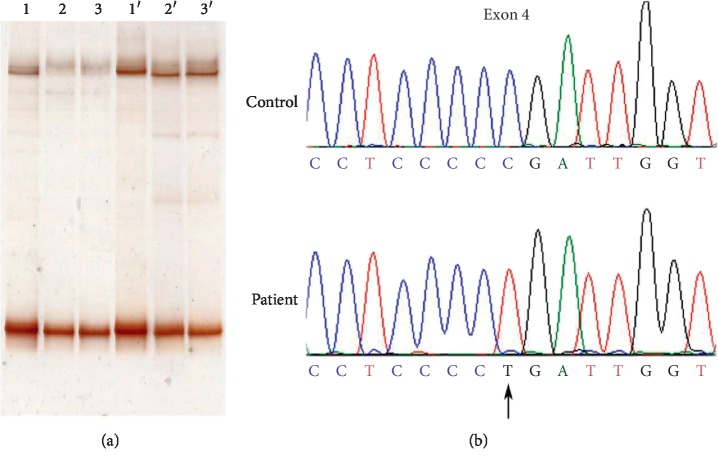
HA, SSCP, and DNA sequence analysis of exon 4 of the *ALB* gene of the patient reported herein. (a) Heteroduplex analysis (HA) and single-strand conformational polymorphism (SSCP) analysis. The DNA sequences encompassing exon 4 and the exon–intron junctions from the patient and two controls were amplified using primers A07A and A08A, and the fragments were electrophoresed on a nondenaturing polyacrylamide gel: lane 1, proband; lanes 2 and 3, and controls. The same samples were denatured and cooled before loading: lane 1′, proband, lanes 2′ and 3′, and controls. (b) DNA sequence of the mutated region of exon 4 in the patient. The arrow indicates the homozygous C-to - transition at position c.412 (c.412C > T).

Because low serum albumin can have causes other than CAA, the clinical diagnosis of this disorder requires confirmation by mutation analysis of the *ALB* gene [1]. Genomic DNA from the proband and from two unrelated healthy volunteers, used as controls, was extracted from whole blood, as described by Watkins et al. [11]. The other family members refused consent for the genetic study. The 14 coding exons of *ALB* and their intron–exon junctions [12] were PCR-amplified from genomic DNA using specific primer pairs [11]. All the 14 coding exons of the *ALB*, encompassing the intron–exon junctions, were amplified, and the products were screened for variations by single-strand conformational polymorphism (SSCP) and heteroduplex analysis (HA). The only differences identified by heteroduplex and SSCP analysis were detected in the 381 bp PCR product obtained with primers A07A and A08A, encompassing exon 4, and the intron 3–exon 4 and exon 4–intron 4 junctions (Figure 3(a), lanes 1 and 1′). The electrophoretic behavior was identical to that previously reported for a boy of nonconsanguineous Swiss parents [13]. The variant region was submitted for DNA sequence analysis, which identified a C-to-T change at position c.412 (c.412C > T) in the homozygous proband (Figure 3(b)). This alters the codon CGA encoding Arg 114 to a stop codon, TGA, resulting in premature termination and is, therefore, responsible for the analbuminemic trait. The putative protein product (p.Arg138Ter) should consist of only 113 residues, but it could not be isolated from the patient's serum. The same molecular defect has been previously reported to cause analbuminemia in two unrelated individuals: the above-mentioned Swiss boy [13] and an American woman [14].

## 3. Discussion

The analbuminemic patient reported here presented with a streptococcal skin infection, which was first treated with amoxicillin/clavulanic acid, later in combination with clindamycin, still later with piperacillin/tazobactam, and finally with oral moxifloxacin. At admission, capillary glucose measurement revealed marked hypoglycemia, with a level of 1.7 mmol/l. Several hypoglycemic glucose levels were documented in the following days of hospitalization (Figure 1), but neuroglycopenic symptoms never occurred. When the antimicrobial therapy was switched to moxifloxacin, recurrent hypoglycemia persisted with a minimum of 2.8 mmol/l (Figure 2). Hypoglycemia has never been reported in patients with CAA in the literature so far. Several mechanisms causing recurrent hypoglycemia in this patient can be discussed. First, the inflammatory state, with a high metabolic rate and prolonged fasting, likely depleted her glycogen stores. In this situation free fatty acid oxidation plays an important role for energy homeostasis. Disorders of free fatty acid metabolism are associated with occurrence of hypoglycemia [15]. Studies from analbuminemic rats have shown improved insulin sensitivity, increased insulin secretion, faster depletion of liver glycogen stores during fasting, free fatty acid deficiency with reduced rates of hepatic lipogenesis, triglyceride secretion, and limited lipoprotein lipase activity [4, 5]. It is unclear if these abnormalities can contribute to the occurrence of hypoglycemia under certain conditions in humans, but an association of hypoglycemia and low albumin levels has been reported for inpatient medical patients recently [16, 17]. Later in the course of this case, antimicrobial treatment with moxifloxacin may have induced hypoglycemia by increasing endogenous insulin secretion. There was no therapy with any other hypoglycemic drug. Further there was no history of gastric surgery like roux-en-Y gastric bypass that is associated with endogenous hyperinsulinism. The period between hospital admission and last sensor glucose measurement was about 4 months. The clearly lowest glucose levels were documented at the time of hospital admission. At least at this time point, a simple connection of hypoglycemia to inflammation and critical illness cannot be excluded. Unfortunately the levels of free fatty acids or ketone bodies were not measured when our patient first presented to the hospital after prolonged food deprivation. As neuroglycopenic symptoms never occurred and the patient reported well being after recovery from the cellulitis, she was not willing to undergo further evaluations for hypoglycemia such as a 72-hour fasting test.

Giving the rarity of CAA and possible other causes of low albumin levels, sequencing of the *ALB* gene should always be performed to confirm a suspected diagnosis. Mutation analysis confirmed the diagnosis of our patient, which was based on clinical and laboratory findings obtained 19 years ago. The cytosine mutated in our case is in a CpG sequence, which was supposed to be a hypermutable site in the *ALB* gene [1]. This molecular defect, c.412 C > T, was previously identified in two unrelated analbuminemic subjects [13, 14]. A different variation at that position, c.412C > G, is known to cause albumin Yanomama (p.Arg138Gly), an alloalbumin present in polymorphic (>1%) frequency in an Amazonian Indian tribe [18]. Our finding of a fourth mutation at that cytosine residue confirms the hypothesis of a high tendency of this region to variation.

We report the first case of recurrent hypoglycemia in a female patient with known CAA. It remains to further investigations if metabolic abnormalities associated with analbuminemia can contribute to occurrence of hypoglycemia. Furthermore, this case demonstrates possible consequences of altered pharmacokinetics in the presence of low albumin levels, which in our case likely caused drug-induced hypoglycemia.

All data generated or analyzed during this case report study are included in this published article. The genetic studies described in this case report were performed in accordance with the ethical principles of the Declaration of Helsinki.

## Figures and Tables

**Table 1:** Biochemical parameters.

Parameter	Result	Reference range
*(a) Baseline*		
Albumin	10	35–52 g/l
Total plasma protein	59	66–87 g/l
Alkaline phosphatase	145	35–105 U/l
Serum cortisol (1600 h)	238	74–286 nmol/l
Thyroid-stimulating hormone	3.58	0.27–4.20 mU/l
Free triiodothyronine	3.8	3.1–6.8 pmol/l
Free thyroxine	12.5	12–22 pmol/l
Gamma glutamyltransferase	52	6–42 U/l
Alanine aminotransferase	31	<35 U/l
Glycated hemoglobin (HbA1c)	4.4	<6.5%
Creatinine	63	45–84 *μ*mol/l
Ionized calcium	1.18	1.15–1.29 mmol/l
Phosphorus	0.91	0.87–1.45 mmol/l
25-OH vitamin D3	9	75–220 nmol/l
Immunoglobulin A	9.63	0.7–4.0 g/l
*(b) After 10 hours of fasting*		
Total cholesterol	9.66	<5.2 mmol/l
High-density lipoprotein cholesterol	1.84	>1.0 mmol/l
Low-density lipoprotein cholesterol	8.05	<2.59 mmol/l
Triglycerides	1.31	<2.0 mmol/l
Free fatty acids	0.24	0.10–0.45 mmol/l
Acetoacetic acid	24	<150 *μ*mol/l
3-hydroxybutric acid	27	<120 *μ*mol/l
Glucose	4.1	3.9–5.5 mmol/l
Insulin	3.6	3.0–25.0°mU/l
C-peptide	408	270–1,270°pmol/l
HOMA^1^	0.7	<2.5

^1^Homeostasis model assessment (Matthews et al. 1985).
